# Familial vulnerability to an unusual cognitive adverse effect of topiramate: Discussion of mechanisms

**DOI:** 10.4103/0019-5545.70986

**Published:** 2010

**Authors:** Chittaranjan Andrade, Savita G. Bhakta, Praveen P. Fernandes

**Affiliations:** Department of Psychopharmacology, National Institute of Mental Health and Neurosciences, Bangalore, India; 1 Department of Psychiatry, Yale School of Medicine, New Haven, CT, Omaha, NE, USA; 2 Creighton University School of Medicine, Omaha, NE, USA

**Keywords:** Cognitive impairment, genetic vulnerability, topiramate

## Abstract

**Background:**

Some patients experience cognitive disturbances with topiramate.

**Case histories:**

A 19-year-old bipolar woman and her 46-year-old mother with paranoid personality disorder both used topiramate (25-50 mg/day) off-label for weight loss. Both women suffer from learning disorders, and both are excessively sensitive to the sedative adverse effects of psychotropic medications.

**Results:**

Within days of starting topiramate, the women began to exhibit troublesome word- and phrase-repetition and word substitution, both occurring only in their written expression. The symptoms were associated with mild sedation, persisted during two weeks of topiramate treatment, and remitted days after topiramate was withdrawn.

**Discussion:**

The presence of the learning disorders and the sensitivity to the sedative adverse effects of drugs may explain why cognitive adverse effects, known to occur with topiramate, developed at the low dose of 25-50 mg/day. The proclivity of topiramate to affect language functions and a possible familial vulnerability herein may explain why the women explained similar, language-specific symptoms. An investigation of topiramate-induced cognitive impairments in family members with epilepsy may throw light on the subject.

## INTRODUCTION

Topiramate is a newer antiepileptic drug which has been approved (as monotherapy or as adjunctive therapy) by the USA Food and Drug Administration for the treatment of partial as well as primary generalized seizures in adults as well as in children above the age of two. Topiramate has also received approval for the prophylaxis of migraine. Other indications for which topiramate may be effective include neuropathic pain syndromes,[1] alcoholism but not necessarily smoking,[2–7] obesity,[8] eating disorders,[9] and drug-induced weight gain.[10,11] Although efficacy in posttraumatic stress disorder is uncertain,[12] the drug is ineffective in bipolar disorder.[13,14]

Broad-spectrum cognitive disturbances are prominent among the adverse effects reported with topiramate.[15,16] For example, Gomer *et al*[17] described deficits in cognitive speed, verbal fluency, and short-term memory in focal epilepsy patients who received topiramate as compared with those prescribed levetiracetam. Such deficits are dose-dependent, occur in up to 44% of the patients even within a dose range of 50-100 mg/day, and reverse upon discontinuation of the drug.[18] Verbal functions appear particularly sensitive to topiramate.[19] As with adults, children are also affected;[20] in fact, topiramate-induced severe but reversible language regression in children and adolescents has also been described.[21]

The risk of cognitive adverse effects with topiramate is probably smaller when the upward dose titration is slow.[22] However, word-finding difficulty, which occurs in about 7% of epileptic patients treated with the drug, may be independent of the titration schedule and may be related to a specific biological vulnerability in the left temporal lobe.[23]

We herein describe an unusual cognitive adverse effect of topiramate in a mother–daughter pair and consider the possible biological underpinnings thereof.

## CASE REPORTS

Ms. N is a 19-year-old female student with a seven-year history of bipolar II disorder. She was receiving bupropion 300 mg/day, escitalopram 20 mg/day, and lamotrigine 300 mg/day. She was much distressed at being overweight (body-mass index=28.2) and requested for assistance with weight loss. Accordingly, she was prescribed topiramate 25 mg/day along with a regimen of diet and exercise; the dose of topiramate was raised to 50 mg/day after a week.

Within days of onset of topiramate treatment, she developed frequent and uncharacteristic word repetition and word substitution. These symptoms were evident only in her written expression. She also complained of impaired creativity; otherwise, there was no impairment in thought processes or in communication functions.

The 46-year-old mother of the patient also wished to lose weight and *suo moto* commenced topiramate 25 mg/day. Within days, she too developed uncharacteristic word-repetition, and also phase-repetition, in her written expression. The symptoms were pronounced and appeared at least once in every two-to-three lines of typed text. Samples of sentences excerpted from the writings of the mother and daughter are provided below.

### In the 19-year-old bipolar proband

#### Word repetition:

“It varies both both horizontally and vertically.”

“I walked through the the mall.”

“The sun’s rays rays stream across the earth….”

#### Word substitution:

“Did to [you] ask the tailor about my clothes?”

“I said [read] an interesting article.”

“I held the pen [stem] of the rose….”

“I know that I make arrows [errors] when I type.”

“I’ve been relatively should [good] this week.”

### In the 46-year-old mother

#### Word and phrase repetition

“Please instruct the client to buy to buy and install the software directly into his system.”

“I am in the process process of obtaining a quotation.”

“Requested from you a year ago from you….”

“I wanted to see to see ….”

Neither woman had ever experienced such symptoms earlier. Neither woman knew of the presence of the symptoms in the other. The symptoms came to light only when the daughter spoke of them during a two-week follow-up visit and the mother, present at the consultation, related her experiences. Mild sedation was the only other adverse effect of topiramate that the women experienced.

Topiramate was discontinued, and both women experienced complete recovery of cognitive functioning and performance within a few days of discontinuation.

Besides bipolar disorder, the daughter suffers from a disorder of written expression that is mild in magnitude; the specific symptom is that she makes frequent mistakes in spelling and punctuation that are markedly out of proportion to her above-average intellectual skills. The mother, who is a high-achieving management consultant, has a mathematics disorder that is mild in intensity. The mother also has spatial learning deficits: she finds it hard to learn directions, and often takes months to become familiar with routes that she travels along frequently. In both mother and daughter, the symptoms of learning disorder date back to childhood and have not been precipitated by any known environmental factor. All diagnoses are based on DSM-IV.[24]

One other matter that may be of relevance is that the mother and especially the daughter are both exceptionally sensitive to the sedative adverse effects of psychotropic medications such as the benzodiazepines and zolpidem (mother) and tricyclic antidepressants, quetiapine, and zolpidem (daughter).

## DISCUSSION

Topiramate has a broad spectrum of action, which may be why cognitive impairments appear more common with this drug than with other antiepileptic agents.[15,25–27] Topiramate increases GABAergic neurotransmission and inhibits glutamatergic neurotransmission; the latter, by blocking AMPA and kainate receptors, and sodium and calcium channels. Topiramate also inhibits carbonic anhydrase.[28–30] Facilitation of inhibitory and inhibition of excitatory neurotransmission probably explains the sedative action of topiramate; sedative drugs are known to impair attention and concentration, and thereby result in downstream impairments of other cognitive processes, as well.

Glutamatergic processes underlie learning and memory through the modulation of synaptic plasticity;[31] therefore, inhibition of glutamatergic neurotransmission may further explain topiramate-induced cognitive deficits. In a separate context, Ojemann *et al*.[32] suggested that a sulfa moiety may also be responsible; topiramate and zonisamide both contain sulfa moieties and inhibit carbonic anhydrase (though, the latter action with topiramate is weak[33]); zonisamide, similar to topiramate, impairs cognition, and verbal functions may be particularly vulnerable.[34,35]

The daughter–mother pair, whom we report, experienced a similar and unusual spectrum of cognitive disturbances with low-dose topiramate: word/phrase repetition and word/phrase substitution, both occurring only in written expression. Each had experienced these disturbances without knowing the existence of the disturbances in the other. Neither had experienced similar symptoms earlier, implying that the mild, topiramate-associated sedation was unlikely to, in itself, explain the symptoms.

### Issues that arise are:

1. Why did these cognitive disturbances arise at such a low dose of topiramate?
2. Why were these unusual cognitive disturbances similar in the daughter and mother?

### Sensitivity to the cognitive adverse effects of topiramate

Topiramate is generally used in target doses of 200-400 mg/day, but doses as high as 1000 mg/day have been prescribed.[36] Although the cognitive adverse effects of the drug may be dose-dependent,[18] the sensitivity to these adverse effects (that is, the development of cognitive adverse effects at low drug doses) may be idiosyncratic; for example, we had earlier reported a patient in whom a 25 mg/day dose of the drug resulted in word-finding difficulty, difficulty in maintaining a stream of thought, and confusion.[37]

We suggest that the daughter and mother whom we report in this article share a common biological and likely genetic explanation for the sensitivity to the cognitive adverse effects of topiramate. One reason to suspect such a common vulnerability is that both suffer from learning disorders; such disorders may have genetic underpinnings.[38,39] The other reason to suspect a common vulnerability is that both are exceptionally sensitive to the sedative adverse effects of psychotropic drugs; whereas, this may be due to a vulnerability associated with the learning disabilities, it may also be due to a genetically-determined inadequacy in drug metabolic capacity. In this context, sedation is a known and common adverse effect of topiramate.[40] Whichever reason applies, the implication is that, in such vulnerable subjects, the cognitive adverse effects of topiramate may appear at lower than usual doses.

With regard to possible topiramate sensitivity associated with language disability, topiramate-induced cognitive impairments in epileptic patients have been suggested to arise in the background of brain dysfunction in areas that process language. For example, functional magnetic resonance imaging in epileptic patients with topiramate-induced cognitive language dysfunction showed significantly decreased activation of the language-mediating regions of the prefrontal cortex, relative to control epileptic subjects.[41] What is uncertain, however, is whether these findings in the imaging describe the pre-existing deficits or deficits inducted by topiramate. With reference to the latter possibility, Cappa *et al*.[42] reported a patient with complex partial seizures who developed non-fluent aphasia with topiramate. An interictal single photon emission computed tomography (SPECT) study in this patient showed focal hypoperfusion of the left lateral and mesial frontal cortex. This finding was no longer evident after withdrawal of topiramate, when language functioning recovered.

### Specificity of the experienced cognitive adverse effects of topiramate

The occurrence of specific cognitive impairments, such as word-finding difficulty,[23,43] word substitution, and word repetition (the cases whom we report) suggests a preferential action of topiramate upon specific neuroanatomical or neurophysiological substrates involved in language. What might these substrates be? In a prospective study of 431 consecutive epileptics prescribed topiramate, Mula *et al*.[23] observed that 7.2% of the patients developed word-finding difficulties. The adverse effect was independent of the dose titration schedule but was, instead, significantly associated with simple partial seizures and a left temporal epileptic focus. Parts of the left temporal lobe are known to be associated with language functions.[44,45]

The occurrence of unusual and strongly similar impairments of written expression in daughter and mother suggests a shared genetic vulnerability with a possible neuroanatomical or neurophysiological basis. Whether the learning disorders (along with the sensitivity to the sedative adverse effects of psychotropic drugs) represent the vulnerability or are merely markers thereof is unknown and requires exploration.

### Treatment

In patients who respond to topiramate but who develop minor language dysfunctions with the drug, strategies must be considered which attenuate the language deficits without necessitating discontinuation of treatment. For example, Wheeler[46] reported six patients with migraine and significant topiramate-induced language and cognitive impairments; the impairments attenuated with donepezil 5 mg/day, allowing an uninterrupted use of topiramate.

## CONCLUSION

We suggest that the presence of learning disorders and/or sensitivity to the sedative adverse effects of psychotropic drugs may predispose patients to the experience of cognitive adverse effects at lower than usual doses of topiramate. We also speculate that as language functions are neurologically localized, and as language impairments may be familial, the proclivity of topiramate to impair language functioning may result from a genetic and/or neurological vulnerability; this would explain why the daughter–mother pair whom we report experienced unusual but closely similar adverse effects. We recommend the investigation of topiramate-induced cognitive impairments in family members with epilepsy so that the validity of these speculations can be determined.
